# Cooperation Survives and Cheating Pays in a Dynamic Network Structure with Unreliable Reputation

**DOI:** 10.1038/srep27160

**Published:** 2016-06-02

**Authors:** Alberto Antonioni, Angel Sánchez, Marco Tomassini

**Affiliations:** 1 Faculty of Business and Economics, University of Lausanne, 1015 Lausanne, Switzerland; 2 Grupo Interdisciplinar de Sistemas Complejos (GISC), Departamento de Matemáticas, Universidad Carlos III de Madrid, 28911 Leganés, Madrid, Spain; 3 Instituto de Biocomputación y F sica de Sistemas Complejos (BIFI), Universidad de Zaragoza, 50018 Zaragoza, Spain; 4 Institute UC3M-BS for Financial Big Data, Universidad Carlos III de Madrid, 28911 Leganés, Madrid, Spain

## Abstract

In a networked society like ours, reputation is an indispensable tool to guide decisions about social or economic interactions with individuals otherwise unknown. Usually, information about prospective counterparts is incomplete, often being limited to an average success rate. Uncertainty on reputation is further increased by fraud, which is increasingly becoming a cause of concern. To address these issues, we have designed an experiment based on the Prisoner’s Dilemma as a model for social interactions. Participants could spend money to have their observable cooperativeness increased. We find that the aggregate cooperation level is practically unchanged, i.e., global behavior does not seem to be affected by unreliable reputations. However, at the individual level we find two distinct types of behavior, one of reliable subjects and one of cheaters, where the latter artificially fake their reputation in almost every interaction. Cheaters end up being better off than honest individuals, who not only keep their true reputation but are also more cooperative. In practice, this results in honest subjects paying the costs of fraud as cheaters earn the same as in a truthful environment. These findings point to the importance of ensuring the truthfulness of reputation for a more equitable and fair society.

In present-day networked society a great many social and commercial interactions take place on internet^1^. In most instances, such interactions involve people who know each other only through an online identity^2^, without any connection whatsoever in the physical world. This is the case, for example, of internet platforms allowing private sales or exchanges among individuals^3^,^4^. In a different but related setting, a host of internet services and physical businesses (e.g., restaurants, hotels, etc.) rely on their online reputation to attract and keep their customers. Key to all these interactions is the reliability of the knowledge on the interaction counterpart, an issue that generates enormous concern these days due to the mounting evidence of fraud^5^. Consumer review websites such as Yelp or TripAdvisor use sophisticated analysis tools to remove (positive or negative) fake reviews; in fact, a whole new technical sub-field called *Online Reputation Management* dealing with how to detect, avoid, and eliminate fake reviews in online sites has recently arisen^6^,^7^. These concerns are even more pressing when personal identities, whose reliability is not externally checked, are the only available information about a possible interaction partner.

In this paper, we address this issue by framing the question in a simplified environment as a dyadic Prisoner’s Dilemma (PD)^8^,^9^ which lends itself to an experimental approach. Indeed, in online exchanges such as those described above, the best joint outcome obtains when both parties involved meet their end of the bargain, but both of them have clear incentives to cheat. In this situation, the game-theoretical prediction picks out defection as the rational choice in this game, but cooperation is often observed in our society and, in particular, in online exchanges. To explain this apparent paradox, several mechanisms have been proposed (see^10^ for a recent review), most of which rely on some form of positive assortment between cooperators^11^, i.e., cooperators interact with individuals of similar behavior and avoid cheaters. In this context, both theoretical models^12^,^13^,^14^,^15^,^16^ and recent experiments with human subjects^17^,^18^,^19^,^20^,^21^,^23^ have established that cooperation may evolve to a remarkable degree when individuals control with whom they interact. Crucially for our present purposes, the process depends on the availability of information on current and possible partners, which subjects then use to evaluate reputation^20^,^21^,^23^,^24^,^25^,^26^,^27^,^28^,^29^ and to decide on their connections. It is then clear that the cooperation-promotion mechanism can act only if reputation scores are truthful, really reflecting the actual individual’s record and are not manipulated in any way.

Here we contribute to the research on fake reputation and its effects by carrying out a controlled experiment^30^ using a PD experiment with the possibility for participants to modify their behavior record by paying a cost. Such cost represents the effort that has to be done to pay or convince somebody else to alter our reputation in order to appear better than that we actually are or to decrease the reputation of a competitor. We could also have considered a cost-free alteration of one’s reputation but, this fact being common knowledge in the experiment, it would have made the concept of a reputation almost useless. Our setup allows us to study whether having individuals with fake reputations around can undermine the evolution of cooperation and the success of dyadic online exchanges. This experimental approach, which to the best of our knowledge has not been attempted before, complements nicely the work carried out from the viewpoint of analyzing fraud evidence and associated behaviors in real systems^31^. As we will see, our results provide new insights on how people behave when they have the possibility to cheat and what are the consequences for the group: Thus, we will show that cooperation is not suppressed by the presence of individuals with fake reputation, but the society splits in two groups, one of them exploiting the other by cheating, leading to a sizeable increase in global inequality.

## Experimental setup

In our experimental sessions, seven groups of twenty subjects connected in a social network played a Prisoner’s Dilemma (PD) game^8^,^9^ with their neighbors. In this two-person game, players must decide whether to cooperate (C) or to defect (D) and, similarly to several recent experimental settings (e.g.^17^,^18^,^19^,^21^,^23^), the chosen action is the same with all neighbors. Note that if actions could be chosen independently for each neighbor the network disappears, and the system is simply a collection of independent pairwise games. If both agents cooperate, each receives a payoff *R*. If one defects and the other cooperates, the defector receives *T* and the cooperator receives the payoff *S*. If both defect, each receives *P*. Since *T* > *R* > *P* ≥ *S*, defection is a dominant strategy and a rational payoff-maximizing player will choose to defect, although mutual cooperation yields a higher collective payoff, whence the dilemma. Subjects played a weak PD game (*P* = *S*) with their immediate neighbors with *T* = 10, *R* = 7, *P* = 0, and *S* = 0. Payoff values are the same as those used in^21^,^22^, where it was shown that when the game is played on a static network cooperation decays, while the possibility to rewire links allows for its emergence and stability when information about past actions of others, which amounts to their reputation, is available. The initial set of connections between the participants was chosen to be a regular lattice of degree 4. Participants played 30 rounds of the sequence described below, although this exact number was unknown to them; they were only told that they would play for a number of rounds between 20 and 50 and without showing them the current round number.

Here, the reputation of a player is expressed through a *cooperation index α* which is the number of times the player has cooperated in the last five moves, thus *α* ∈ [0, 5]. We considered two treatments: a baseline one, called *Real Reputation* (RR) in which the cooperation index cannot be manipulated, and a modified one in which participants were informed that all of them were allowed to vary their cooperation index by paying a cost, called *Fake Reputation* (FR). At the beginning, all players receive an initial *α* of 3 based on the actions sequence *CDCDC*. Note that this form of reputation is related to but different from the one used in^21^,^23^. While in those earlier studies explicit past choices of each player were available to all others, in our experiment, there is some uncertainty about the current behavior of a player even in the RR treatment. This uncertainty comes about because only the number of cooperative actions of the current first neighbors and potential partners is known, but not their order. In addition, neighbors are just unlabeled anonymous individuals who cannot be recognized from one round to the next. In this respect, it is worth noting that most of the reputation subjects assign to partners arises from their average cooperativeness without reference to the chronological set of actions^21^. On the other hand, this is also the case in many e-commerce platforms (e.g., Amazon) where only an average success rate of interactions with external sellers is provided. In this sense, our setup reproduces a real-world situation in which a subject interacts with a partner for the first time, i.e., when first-hand information about the partner is not available.

In the RR treatment each round consisted of the following four stages:

1. Action choice
2. Neighborhood modification
3. Link acceptance decision
4. Feedback on payoffs

In the first stage, players receive information on the cooperation index of their current neighbors and have to select one of two actions, *A* or *B*, where *A* implied “cooperation” and *B* implied “defection”, the action being the same with all neighbors, as said above. We chose to label actions in a neutral fashion to prevent framing effects^32^,^33^. In the second stage, participants may decide to unilaterally suppress a link with a neighbor and they are also given the option to offer a link to a new, randomly chosen partner; in both cases, they only know the *α* value of the corresponding subject. In the following stage, participants see all link proposals from other players (and their *α*), which they can either accept or reject. After these decision stages a new network is formed, and subjects accumulate their payoff by playing the PD game in pairs with their current neighbors. They are neither informed about their neighbors’ payoffs nor about their neighbors’ individual current actions. Participants never know the full network topology.

The FR treatment is identical to the RR treatment with the following fundamental difference: Participants never know whether the observed cooperation index *α* of their partners is the real one or has been modified. Consequently, in this setup there is an additional stage between stages 1 and 2 of the RR treatment during which participants may choose to pay a cost in order to modify their *α* value. The chosen cost was 4 points for each unity of reputation modified, per round. For example, if a player has currently an *α* value of 2 based on her actual last five actions, she can decide to pay 8 points to show an *observable cooperation index* of 4 to the partners. This modification only lasts for the current round. If a player wants to change her observable *α* again for the following round she has to pay the cost anew. Apart from that, as in the RR treatment, there is no cost implied if one just wants to show her true cooperation index. Before choosing the above value of four for the cost we performed a preliminary laboratory session in which the cost was set to nine points instead. In that case, we observed that very few players chose to pay that cost to modify their observable *α*. Conversely, if the cost is too small then the players would cheat too frequently which would make the cooperation index signal almost useless.

We performed the RR treatment six times where three groups of 20 participants performed the same experiment two times each. The FR treatment was run eight times by four groups each playing two times. Before each new session, we re-initialized the regular lattice by reshuffling the participants who played the same experiment in the same treatment condition for other 30 rounds.

## Results

**Figure 1:**
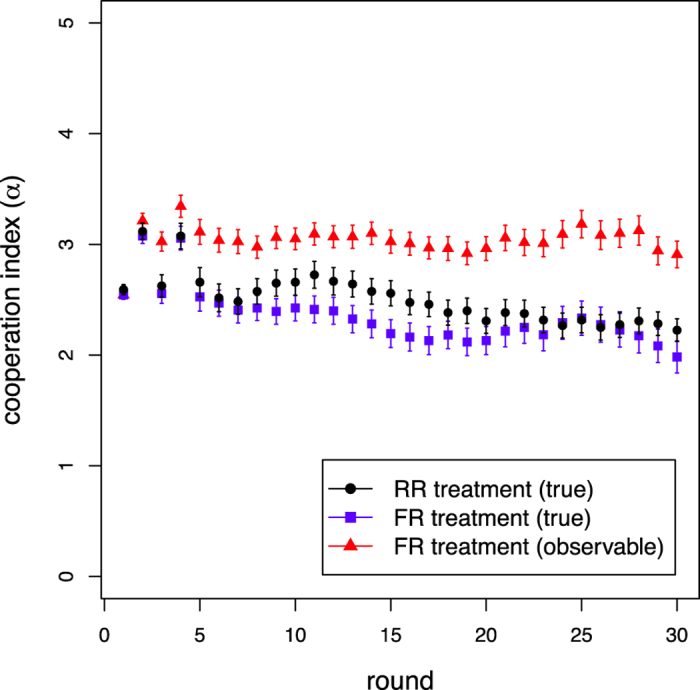
True and observable cooperation index *α* in the whole population aggregating all treatments in the baseline case (RR, black dots) and the case with fake reputation (FR, blue squares). The observable *α* in the FR treatment is represented by the red triangles. Error bars represent standard errors of the mean. The difference between final mean values of true *α* is not statistically significant [first repetition, *P* = 0.416; both repetitions, *P* = 0.336]. The difference between final mean values of RR true *α* and FR observable *α* is statistically significant considering both repetitions [first repetition, *P* = 0.138; both repetitions, *P = 0.019].

We now turn to the discussion of our experimental results. First, we look at the behavior of the average cooperation index *α* for the baseline case (RR) and for the fake reputation case (FR), see Fig. 1. The time evolution of cooperation in the population, which is noisier, parallels that of *α* and it can be found in the SI, Fig. S1.

We now compare the aggregate cooperation frequency results with those obtained in similar recent experimental studies^17^,^18^,^19^,^20^,^21^,^23^. However, one must bear in mind that, although the settings are similar in the sense that participants can cut or form links at different rates, the details differ either in link updating frequency, partner accepting rules, information available to the players and, most importantly, the PD payoff matrix values used. In Rand *et al.*^17^, the “fluid dynamic network” treatment is similar to ours, although links to cut and to create are randomly chosen and presented to the players. The information set is also different: the focal player knows the last action of the player at the other end of a random link. In these conditions, Rand *et al.* find that the cooperation frequency stays around 0.6 during all rounds. In Wang *et al.*^18^ players update their links at various rates. Information consists in the knowledge of the last five moves of all players. Cooperation stays high at the beginning (more than 0.8) for almost all update frequencies and tends to decay in the final rounds. This behavior is rather expected since this is the only study among those mentioned in which the participants know the exact number of rounds and they are thus eager to defect in the last ones. In Antonioni *et al.*^20^ information on the last action of a potential neighbor is costly to participants and it strongly influences the outcome of the experiment. In fact, final cooperation frequencies oscillate between 0.4 and 0.6 for the two values of the cost. On the other hand, when this information is costless cooperation frequency can reach 0.8–0.9. In Cuesta *et al.*^21^ the authors investigate how the amount of reputation available influences cooperation in a dynamical environment in which unwanted links can be cut and new ones formed in a manner qualitatively similar to all previously described settings. Reputation is given by the sequence of the last *m* actions of any given player where *m* can be varied between 0 and 5. The authors find that there is a clear positive correlation between *m* and the cooperation level. For *m* = 0 cooperation quickly decays from an initial 0.5 to 0.2 at the end of the runs. On the other hand when *m* > 0 cooperation is sustained with *m* = 3 and *m* = 5 giving statistically indistinguishable results with a roughly constant cooperation level between 0.5 and 0.6. In Gallo and Yan^23^ there are four treatments which differ in the amount of information participants have about their partners and about the whole network. In the baseline treatment subjects only know the previous five actions of their direct neighbors, while in the most information-rich environment they know the previous five actions of all players, as well as the topological structure of the current network. The remaining two settings are in between the previous ones. Concerning the level of cooperation, they found that global reputational knowledge is the main determinant for the sustenance of cooperation, which stays at about 0.5–0.6 over the whole period. Knowledge of the structure of the whole network does not help. By contrast, in the setting in which reputational knowledge is only local cooperation stays at about 0.3. Finally, in Fehl *et al.*^19^ cooperation reaches high levels around 0.7 but their setting cannot be compared with ours, nor with the above ones because agents there can choose a different action with different neighbors.

With respect to the above-mentioned studies where cooperation is high and remains stable in dynamical networks when information about the partners’ strategy is complete, in our case cooperation is maintained but at a lower level (see also Fig. S1 in the SI). We believe that the reason for this difference is to be found in the higher level of uncertainty. Even when *α* cannot be faked (RR treatment), the single index that people see being an average and not the true temporal sequence of actions, does not allow cooperative acts to be identified with certainty and participants are left guessing to some extent. In fact, all sessions started with a fraction of cooperators of about 0.6 and this fraction was about 0.5 at the end (see also Fig. S1 in the SI). On the other hand, as shown in^21^, knowledge of the last action of possible partners plays an important part in reputation assignment, going from almost 30% when information comprises the last 3 actions to more than 16% with 5 actions. This missing piece of information may lead subjects to estimate their counterparts’ reputation to be lower than what they would do with more information, and therefore to decrease their cooperativeness. Whatever the case, it is important to notice that cooperation based on this kind of easily manipulable reputation system still seems to be fairly high, although our results are not conclusive about the possibility that it will eventually decay. Hence, at least as far as first interactions are concerned, we did not observe a serious hampering of the willingness to cooperate. Other explanations on the observed cooperative behavior are also possible e.g., the influence of the payoff matrix values^34^,^35^ and group sizes^36^,^37^. Unfortunately, we were not able to run another setting because of time and financial constraints.

Let us now move into between-subject differences in behavior. To that end, in Fig. S3 (see SI) we analyze the average participants’ frequency of cooperation in deciles for the RR treatment (black bars) and for the FR treatment (blue bars). Interestingly, it can be seen that in the RR treatment about one third of the participants cooperate between 50% and 60% of the times. Such a peak of cooperation is not observed in the FR treatment where the frequencies tend to be more uniform. In fact, in the FR treatment some participants decided to maintain a lower cooperation frequency and to increase their observable cooperation index paying the cost.

**Figure 2:**
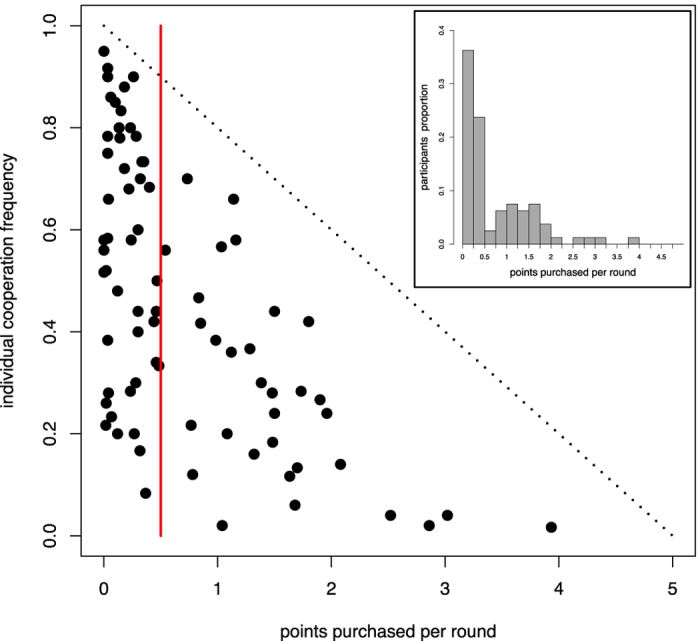
Scatterplot of the participants main behavioral features in the FR treatment. The *x*-axis value is the average number of points that a given player has paid per round while the *y*-axis represents her frequency of cooperation. The red line separates the area containing participants we have called *reliable* (left side) players from the so-called *cheaters* (right side), while the dotted diagonal limits the feasible space a player can be in. Inset: histogram of the proportion of participants who buy a certain amount of points per round on average.

Figure 2 sheds more light on this issue by representing the position of each player in a space where the *x*-coordinate is the player’s average number of points paid per round and the *y*-coordinate is her cooperation frequency for all sessions of the FR treatment. It can be clearly seen that most players cheat only rarely, buying less than half a point per round. Thus we have, somewhat arbitrarily but sensibly, traced the dividing line at this point. As will be shown below, this criterion does reflect very well the two main types of behavior in the population. We have dubbed the players that appear in the area to the left of this red line “reliables”. By contrast, the rest of the players, those who buy more than half a point per round in the average, will be called “cheaters”. Among reliables, we observe a heterogeneous behavior: Some are essentially cooperators (top left corner of the scatterplot), some are mostly defectors (bottom left corner), and the rest have a mixed behavior. Cheaters, on the other hand, cooperate less on average. The inset summarizes this information in an aggregate manner by showing the proportion of players that buy a certain amount of points per round. Reliable players are seen to be around 60% of the total. Most cheaters buy between 0.5 and 2.0 points per round, while very few increase their *α* by more than two points per round. This suggests that most cheaters tend to stick to an observable cooperation index of about three in order not to trigger link cutting from neighbors. Also, we plot the normalized number of players purchasing points per round and per participant type in Fig. S8 of the SI.

**Figure 3:**
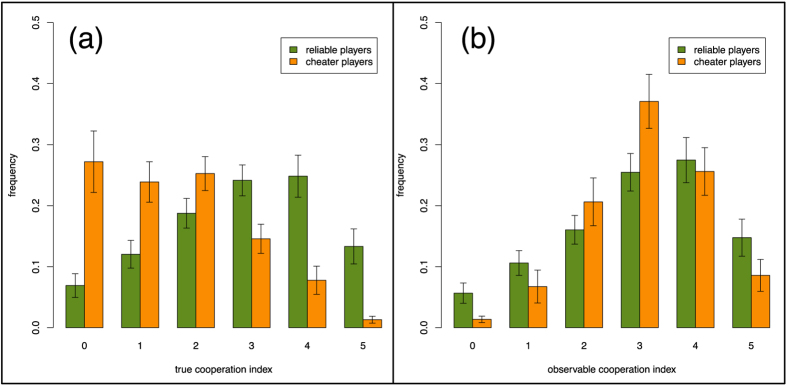
Frequency of experimental cooperation indices in the FR treatment separately for cheater and reliable players and for all treatments and rounds. (**a**) The panel depicts the frequency of the true cooperation index; (**b**) the panel shows the observable cooperation index. Note that, while reliables behave coherently and have similar *α* profiles, cheaters cooperate much less but tend to show an observable cooperation index comparable to that of reliables. The difference in distribution between true cooperation indices is always statistically significant when observed at the beginning and the end of the treatment [first repetition at first round,**P = 0.003; both repetitions, ***P < 0.001; first repetition at last round, ***P < 0.001; both repetitions, ***P < 0.001]. The difference in distribution between observable cooperation indices is never statistically significant [1st at first round, *P* = 0.509; both, *P* = 0.640; 1st at last round, *P* = 0.985; both, *P* = 0.388]. The difference in distribution for reliable players is never statistically significant [*P* > 0.9] while for cheater players is always statistically significant [***P < 0.001] at the beginning and the end of the treatment. Error bars represent standard errors of the mean.

In order to confirm the above hypothesis we plot in Fig. 3 the histograms of the cooperation index in the population for the two categories of subjects. We expect that, if our definition makes sense, reliable players should have very similar true and observable *α*, and this is indeed the case. This does not hold for cheaters, who tend to increase their observable *α* when the true one is 0 or 1 comparing the histograms in Fig. 3a,b. This is quite understandable given the setting of the experiment and supports our interpretation, namely that the participants’ apparent goal seems to be to show a cooperation index of about three, which guarantees a “fair” behavior on the part of the neighbors who will not be tempted to cut their link to them. In this respect, it is important to note that the general appearance of the histogram of observable *α* for cheaters is very similar to that of reliables (Fig. 3b), indicating that cheaters grasp what the acceptable behavior should be. If any, the main difference is that the histogram for cheaters is more peaked around three, i.e., there are fewer cheaters showing a very high *α*, in agreement with our intuition that they do not need to look very cooperative. In our experiment we have noticed that participants severe a link about 20% of the time both in the RR and FR treatments. In this respect, Fig. S6 shows that subjects are quite heterogenous in their link cutting frequency, with a majority of them cutting links less than 20% of the time while others severe their connections much more often (even up to 80% in some cases). From Fig. 3b we can also infer that cheaters use their observable *α* to avoid having their links cut off as they appear to be “reliable”. Their observable *α* also helps them to be accepted by other players in the link proposal phase. This can also be inferred from Fig. S7, that shows that the higher the *α* of an individual, the more likely her acceptance as a new neighbor. Interestingly, in the FR treatment new links have a smaller acceptance rate than in the RR treatment, which is most probably due to the uncertainty about the observable *α*. On the other hand, the differences between reliables and cheaters give rise also to noticeable traces on the aggregate behavior: The cooperation level of reliables is considerably larger than that of cheaters (cf. Fig. S1 in the SI), while their combination mimics the true cooperation index of the RR treatment (cf. Fig. S2a in the SI). Again, the observable cooperation index turns out to be almost the same for both kind of players (cf. Fig. S2b in the SI). It is also remarkable that reliables exhibit a somewhat larger cooperation, as if their honest behavior in terms of reputation would be associated to higher cooperativeness (in a manner not unrelated to the “phenotypes” reported by Peysakhovich *et al.*^38^). We also mention the work by Biziou-van-pol *et al.*^39^ who studied the relation between cooperation, altruism, and aversion to telling white lies. White lies are those that increase the benefit of the liar and/or somebody else. Specifically, the authors find that there is a negative correlation between telling white lies that benefit both the other person and the liar and cooperative behavior. This is in line with our findings but one should bear in mind that our subjects tell so called “black lies”, which are those that increase a person’s benefit at the expense of another.

**Figure 4:**
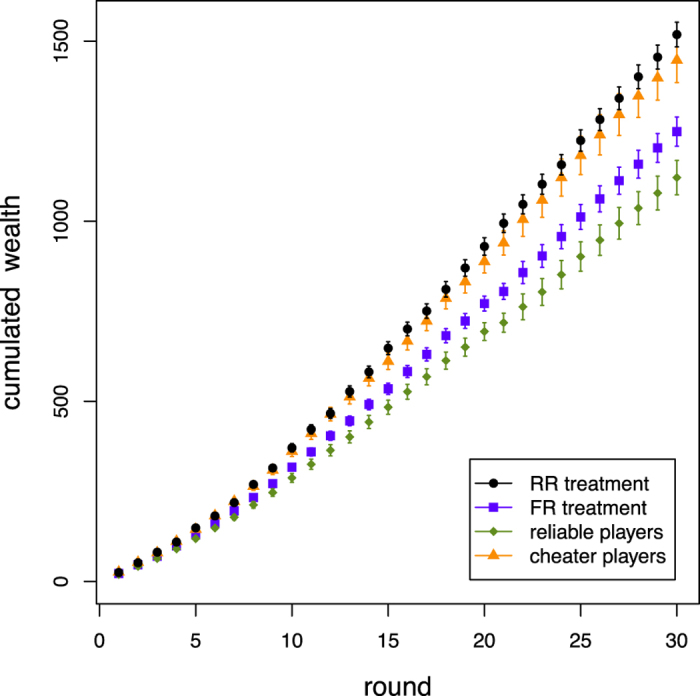
Cumulated participants’ wealth averaged over all sessions for the RR (black dots) and FR (blue squares) treatments. In the latter case we also plot the wealth for reliables (green squares), and cheaters (orange triangles) separately. The cost for reputation modification is taken into account. Error bars represent standard errors of the mean. The difference between final mean values of cumulated wealth for RR and FR treatment is statistically significant considering both repetitions [first repetition, *P* = 0.189; both repetitions, *P = 0.023].

So far, we have reported that while the level of cooperation observed in the two treatments is basically the same, the population in the FR treatment shows a clear splitting in two subpopulations, reliables and cheaters. Does this segregation lead to noticeable consequences at the population level? To answer this question, Fig. 4 shows the experimental average cumulated payoff, or social wealth, by treatment and type of player as a function of the round number. First, we note that participants have the best payoff in the RR treatment (black dots). We interpret this result as being a consequence of three factors: a slightly higher cooperation level in the RR treatment (see Fig. 1), the absence of a cost to increase one’s reputation, and a slightly higher average degree of the players network in the RR case (cf. SI, Fig. S4). Likewise, the blue squares in Fig. 4 reports the cumulated wealth in the FR treatment whereas the green and orange symbols show, respectively, the cumulated gain for reliables and cheaters taking the cost into account. An interesting result is that cheaters gain more than reliables in spite of paying the cost of cheating. Having a higher reputation allows a cheater to maintain and create more connections to neighbors (cf. SI, Fig. S4) which tends to increase her payoff. Furthermore, a cheater tends to defect more often and thus to earn the maximum payoff *T* in many encounters. Thus, although when cheating is possible the total gain is less than in the RR treatment, we see now that it is more profitable to a be a cheater in the FR setting. As a result, the inequality in our “society” increases: The fact that cheaters earn a higher payoff leads to a Gini coefficient of 0.370 in the FR treatment, to be compared to a value of 0.271 in the RR treatment, both indices being calculated on the cumulated wealth of participants at the end of the experiment.

For completeness, we also show the time evolution of the participants’ payoff in the SI, Fig. S5. Now, comparing the cumulated gain of cheaters in the FR treatment with the cumulated gain in the RR treatment shows that there is little difference but the payoff is slightly larger in the latter. Thus, although it pays to be a cheater when faking one’s reputation is allowed, if nobody is allowed to do it the social wealth is higher, at least as far as this experiment is concerned.

## Discussion

In summary, we have designed and carried out an experiment in order to test the effects of uncertainty about the reputation of possible partners in the frame of the Prisoner’s Dilemma game. Our experiment provides us with enough evidence to support several important conclusions. To begin with, the aggregate cooperation level of the population does not change when reputations can be faked. Interestingly, this is in agreement with the only game-theoretical work we know of in this context: Röhl *et al.*^40^ showed, in an evolutionary public goods game, that fake reputation does not harm cooperation under some conditions in well-mixed populations. We must stress that their approach is quite different from ours, in particular because every individual interacts with every other one and cannot modify this interaction neighborhood. On the other hand, Röhl *et al.* introduce a probability to be discovered and punished which is reminiscent of the link-cutting stage of our experiment: Note that our subjects cut links without knowing for sure that the corresponding individual is a cheater, which is not unrelated to probabilistic discovery. Therefore, in spite of the differences, the fact that our observation aligns with the predictions in^40^ is certainly suggestive. Another interesting line of research related to the findings we are reporting here is that of image scoring in evolutionary games^24^,^41^, based on the idea that helping someone increases one’s image score, whereas refusing to help reduces it. This is clearly similar to the notion of reputation, except that when it is kept by each individual separately it becomes private instead of public as we consider here. In this regard, it has been recently shown^27^ that when there is information on group scoring only a few images are needed to sustain reputation. This points to the possibility of preventing the problems of faking reputation by externally providing some manner of (truthful) group information.

Nevertheless, our results go further than this as they point to a splitting of the experimental population in two different types of individuals: reliables, who cheat very little if at all, and cheaters, who are willing to fake their reputation almost at every interaction. This cannot be noticed by looking at the cooperation level and only an analysis of the within-subject variability allows to uncover this effect. In spite of the fact that cheaters have to pay some cost to modify their cooperation index, they still end up making more profit than reliables, as they manage to exhibit an intermediate reputation that makes them less likely targets for link cutting and more likely to be accepted as new partners. The similarity between the histogram of the cooperation index in both treatments is striking and proves that cheaters have a correct intuition about what is the optimum level of reputation modification to do well. In addition, it turns out that the average earnings of cheaters are very similar to the case in which reputation is truthful, which implies that reliables are in practice paying the cost of the cheaters’ efforts to disguise their bad behavior. On the other hand, reliables are honest not only with respect to their reputation, but also about being even more cooperative than the general population in the baseline treatment. As a result of the combination of reliable and cheating behaviors, inequality increases: the Gini coefficient of the RR treatment increases by more than a 30% in the FR treatment; for a comparison which only has illustrative value, these would be values similar, respectively, to Finland in 2008 and Tanzania in 2007^42^. We therefore conclude that, in our experiment, even if the level of cooperation in interactions is basically the same as when reputation is truthful, the features that emerge from the possibility of faking reputations are the splitting into exploiters and exploited, and a larger degree of inequality, both highly undesirable. While this is a first step in the experimental analysis of the problem that cannot be easily generalized to more complex social environments, it is clear that the conclusions are still very relevant and would justify further, intensive research along these lines, in order to inform policy makers’ efforts to ensure fair and transparent trade.

## Methods

The use of human subjects in this experiment has been approved by the Ethics Committee of the University of Lausanne and our methods were carried out in accordance with the approved guidelines. Participants signed an informed consent describing the nature of the experiment before they entered into the laboratory. We conducted a total of seven experimental sessions in November 2014. Participants were recruited from the pool of undergraduate students from all disciplines of the University of Lausanne and the Ecole Polytechnique Fédérale of Lausanne using ORSEE^43^. Subject-subject anonymity was granted at all stages, and the experiment was computerized using the z-Tree environment^44^. Before making decisions, participants read detailed instructions and responded to a set of control questions in order to insure common understanding of the game and the computation of payoffs. A translation of these instructions from the original French is provided in Section S1 of the SI. Each session lasted asted one and a half hours and included 20 participants, where a total of 140 subjects, 48 women and 92 men, took part in the experiment. Participants were randomly assigned to the RR treatment (60 subjects) and to the FR treatment (80 subjects). Subjects observable demographic variables did not qualitatively differ across treatments. Participants received a show-up fee of 10 CHF (about 10.50$), and their final score in points was converted at an exchange rate of 1 CHF = 120 points. The average payoff per subject was 27.35 CHF (about 29$). All statistical difference significances of mean values have been obtained performing unequal variances *t*-test analysis. All statistical difference in distribution have been obtained performing two-sample Kolmogorov-Smirnov test analysis. We considered the fifth one as the first observable and independent round to compare cooperation index distributions. Since each group performed two repetitions of the assigned treatment we considered two different statistical approaches. The first one takes into account independent observations, thus considering only the first repetition of the treatment, while the second analysis assumes both repetitions of the treatment as two independent observations.

## Additional Information

**How to cite this article**: Antonioni, A. *et al.* Cooperation Survives and Cheating Pays in a Dynamic Network Structure with Unreliable Reputation. *Sci. Rep.* **6**, 27160; doi: 10.1038/srep27160 (2016).

## Supplementary Material

Supplementary Information
