# Supplementary material for: Cooperation Survives and Cheating Pays in a Dynamic Network Structure with Unreliable Reputation
Source: Sci Rep. 2016 Jun 2;6:27160. doi: 10.1038/srep27160 (PMC4890023; doi:10.1038/srep27160)
Supplement: Supplementary Information [file srep27160-s1.pdf]

Cooperation Survives and Cheating Pays in a Dynamic Network Structure with Unreliable Reputation

Supplementary Information

A. Antonioni, A. Sánchez, and M. Tomassini

The Supplementary Information (SI) provides translation from French of the exact instructions form that participants received for the FR treatment and corresponding trial questions, see Section 1. Instructions for the baseline RR treatment are identical without the *apparent profile* stage.

Additional results of experimental data that are not shown in the main text are presented in Section 2.

1 Instructions (translated from French)

Welcome to this experiment !

You are going to take decisions that will affect your payoffs as well as the payoffs of the other participants.

Although all payoffs are expressed in number of points, these points will be transformed into money at the end of the experiment according to the following exchange rate:

120 pts. = 1.- CHF

During the experiment it is **strictly forbidden to talk to other participants**. If you have a question, please ask the assistants. If you don't comply with these rules, we will regrettably be obliged to exclude you from the experiment.

In this experiment every participant will interact with his/her "neighbors" in a network constituted by all the participants in the room. At the beginning of the experiment everybody will have four neighbors but this number may change during the experiment as explained below.

During the experiment you'll only see your immediate neighbors, i.e. only the participants with whom you are directly linked; you will not see what happens in the rest of the network (for instance, you won't be able to see what the neighbors of your direct neighbors are doing).

What is it all about?

There will be a number of rounds that is comprised between 20 and 50 but the exact number will not be made explicit. Each round consists of four steps:

1. Decide your action
2. Decide your apparent profile
3. Modify your neighborhood
4. Accept or refuse new links

1. Decide your action

In this first stage you will have to choose an "action" among the two following options:

A or **B**

Like you, your neighbors will also have to take the same decision about the action they will choose. The chosen action is unique: this means that the same action will be used when interacting with all of your neighbors (you can't use different actions with different neighbors). Your payoff for the current round is computed as a function of your current action and the current actions of your neighbors.

Now we explain the payoffs for each possible combination of your action and one's of your neighbors action:

- You choose **A** and your neighbor chooses **A**:
  you gain **7 points** and your neighbor also gets **7 points**.
- You choose **A** and your neighbor chooses **B**:
  you get **0 points** and your neighbor gets **10 points**.
- You choose **B** and your neighbor chooses **A**:
  you gain **10 points** and your neighbor gains **0 points**.
- You choose **B** abd your neighbor chooses **B**:
  you get **0 points** and your neighbor gets **0 points**.

Your final accumulated payoff in each round is computed as the sum of the points gained in each interaction with each of your current neighbors.

However, the relevant neighbors for the payoff computation are those to whom you are directly linked **at the end of each round**. As you shall see below, you will be allowed to modify your neighborhood in the following stages before the round ends.

The following examples illustrate the computation of your payoff at the end of a round. Note that in the examples you are the central player, with your action underlined, and you have **4 neighbors** but during the experiment you could have a different number of neighbors.

Example 1 : Your action is **A**, the action of all your neighbors is also **A**.

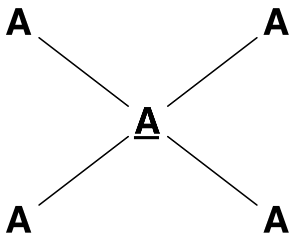

Your payoff is : $7 + 7 + 7 + 7 =$ **28 points**.

Example 2 : Your action is **A**, the action of all your neighbors is **B**.

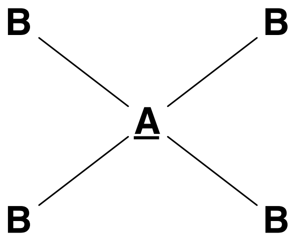

Your payoff is : $0 + 0 + 0 + 0 =$ **0 points**.

Example 3 : Your action is **B**, the action of all your neighbors is **A**.

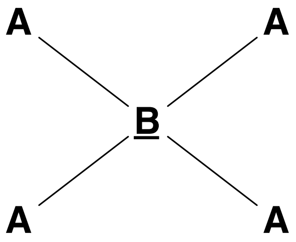

Your payoff is : $10 + 10 + 10 + 10 =$ **40 points**.

Example 4 : Your action is **B**, the action of all your neighbors is also **B**.

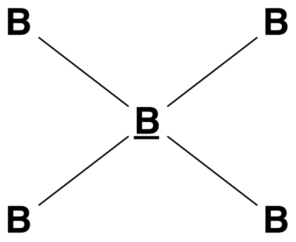

Your payoff is : $0 + 0 + 0 + 0 =$ **0 points**.

Example 5 : Your action is **A**, the action of three of your neighbors is **A** while the fourth one does **B**.

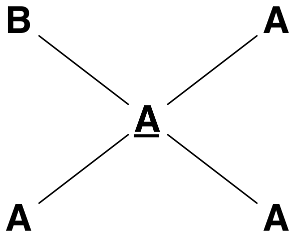

Your payoff is : $0 + 7 + 7 + 7 =$ **21 points**.

Example 6 : Your action is **B**, two of your neighbors do **A** while the other two do **B**.

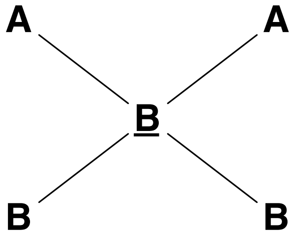

Your payoff is : $10 + 10 + 0 + 0 =$ **20 points**.

The Profile

The *profile* of a participant is computed from the sequence of her/his last five actions. It is defined as the number of **A**s in the sequence and, by consequence, it belongs to the integer interval $[\mathbf{0}, \mathbf{5}]$.

For example, if the past actions sequence is:

A	B	B	A	B

the corresponding profile value is **2**.

If the past actions sequence is:

B	A	A	A	B

then the corresponding profile value is **3**.

The above definition refers to the profile called *real*.

You have the option of choosing an *apparent* profile different from the real one at each round, according to the procedure explained in the next section of this document. This apparent profile is the one that your neighbors will see. The real profile is private and will not be communicated to your neighbors unless your apparent profile is the real one, i.e. you choose not to alter it in the current round.

On the other hand, note that the current actions of your neighbors will never be known to you, only their apparent profiles.

IMPORTANT

Note that at the beginning of the experiment all participants will start with the same initial sequence of actions as follows:

A	B	A	B	A

which amounts to an initial profile value of **3** for all.

During the experiment the profile will be denoted by "P".

In the first stage of each round you must choose the action that you want to use during the current round with the help of the following screenshot:

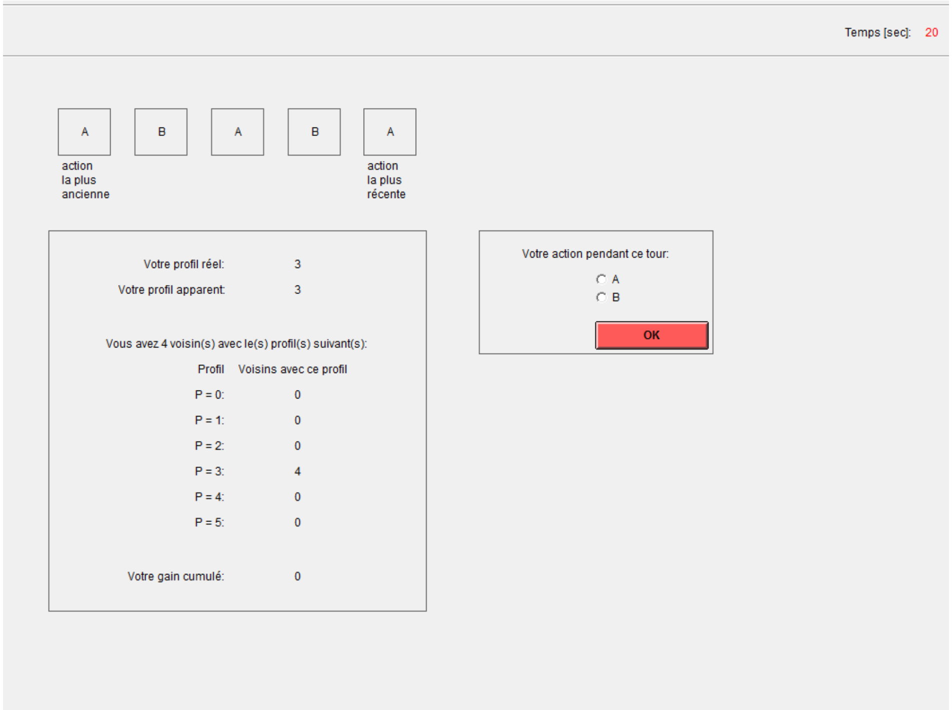

Choose your action for this round and click the "OK" button.

2. Decide your apparent profile

In this stage you must decide what to do with your profile for this round.

Important Note: each point added or subtracted to your real profile will cost you **4** points. For example:

- if your profile real value is **2** and you would like to increase it to an apparent profile of **4**, you will have to pay **2×4=8** points.
- if your real profile is **3** and you want to have an apparent profile of **2** then you must pay **1×4=4** points.

If you decide to keep your apparent profile as your real profile it will cost you **0** points as this is the default value. If you would like to change your apparent profile but you cannot pay for the corresponding points your apparent profile will be the same as your real profile for the round. Also note that during the first round you cannot change your apparent profile since all participants start with **0 points**.

You will choose your apparent profile for a give round through the following screenshot:

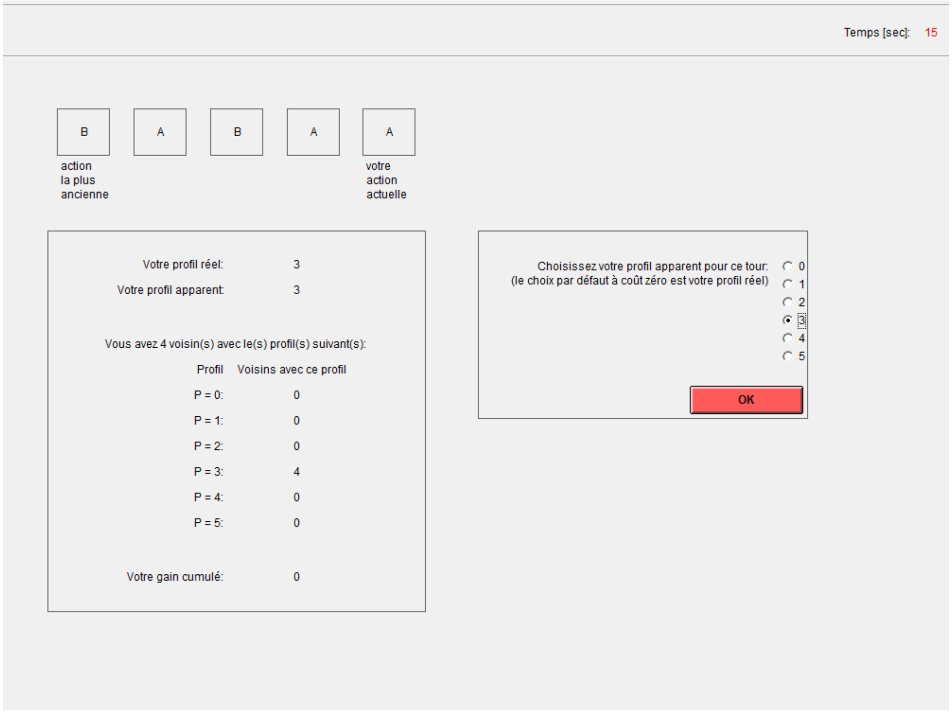

Choose your apparent profile for this round and click the "OK" button. If you want to keep your real profile as apparent profile just click directly the "OK" button.

3. Modify your neighborhood

In this stage you must decide whether you want to modify your neighborhood. You'll see in the screen the number of current neighbors you have as well as the values of their apparent profiles. Next, you must decide the following:

- decide whether you want to cut a link to a neighbor with a certain current apparent profile value. An arbitrary link to such a neighbor will be cut automatically and unconditionally.
- decide whether you want to propose a link to a randomly chosen participant who is not yet one of your direct neighbors. The system will made her/his apparent profile known to you. This link will only be created if the partner will accept it in the next stage. In some cases, it will not be possible to propose you creating a new link.

The possible modification of your neighborhood will be made through the following screenshot:

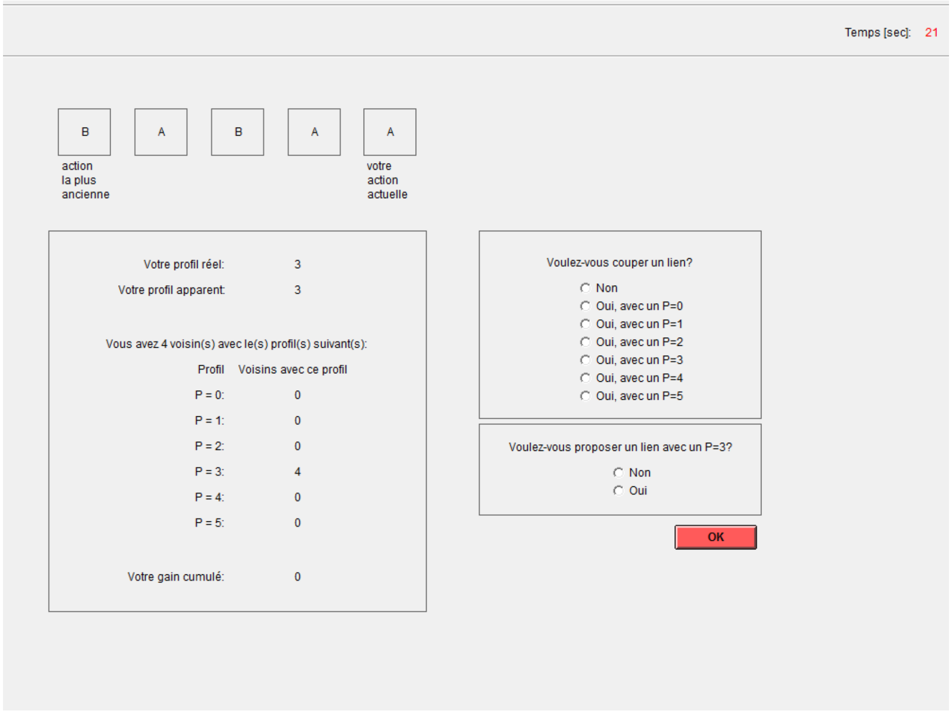

Choose whether you want to cut a link and/or whether you want to propose a new link, then click the "OK" button.

4. Accept or refuse new links

Finally, in this stage you must decide which links, among those proposed by other participants, you are going to accept after knowing the apparent profile of the potential partner. Note that a link will only be created if you accept it at this stage. The proposing participant does already know your apparent profile and decided to propose you creating the link.

You will complete this stage by using the following screenshot:

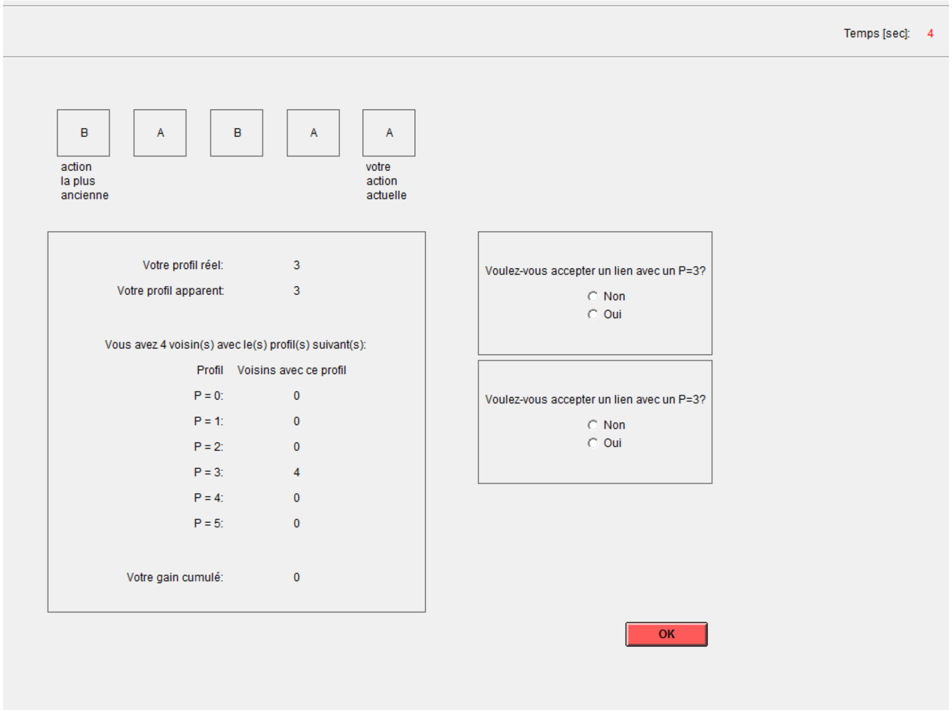

For each link proposal, choose whether you want to accept it then click the "OK" button.

End of round

According to the decisions of all participants, a new network will be created. Your payoff for the round will be computed as a function of your own action and of the actions of all your current neighbors according to the procedure described in the section “Decide your action”. From this payoff value it will be subtracted the cost of changing your apparent profile if you decided to do so in this round.

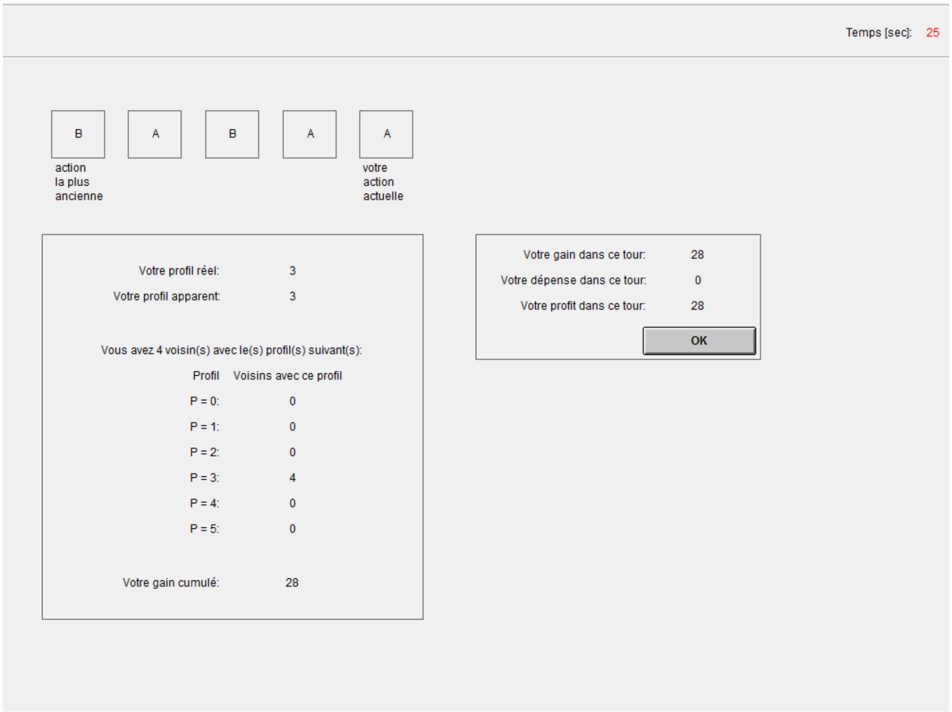

Click the "OK" button to go to the following round.

Did you understand the explanations ?

Before starting the actual experiment we would like to be sure that you and everybody else has correctly understood the decisions that you’ll have to make. To this end, please answer the questions that will appear on your screen. When you are done with a question click the "OK" button at the bottom of the screen.

Trial questions

Participants answer the following trial questions after reading the instructions. Wrong answers were not accepted by the software in order to continue to the following question. Participants were able to begin the experiment only answering correctly to all questions.

1. How many neighbors do you have at the beginning of the experiment?
   Correct answer: 4 neighbors.
2. Suppose that at the end of a round you have 3 neighbors. Your action is **A** and all your neighbors have chosen **A**.
   Which is your gain at the end of this round?
   Correct answer: 21 points.
3. Suppose that at the end of a round you have 3 neighbors. Your action is **B** and all your neighbors have chosen **A**.
   Which is your gain at the end of this round?
   Correct answer: 30 points.
4. Suppose that at the end of a round you have 5 neighbors. Your action is **A** and three of your neighbors have chosen **B** while the other two of them have chosen **A**.
   Which is your gain at the end of this round?
   Correct answer: 14 points.
5. Suppose that at the end of a round you have 5 neighbors. Your action is **B** and three of your neighbors have chosen **B** while the other two of them have chosen **A**.
   Which is your gain at the end of this round?
   Correct answer: 20 points.
6. If you decide to **cut off** a link, is the link automatically cut off or is it necessary that also your neighbor accept this decision?
   Correct answer: The link is automatically cut off.
7. If you decide to **create** a link, is the link automatically created or is it necessary that also your neighbor accept this decision?
   Correct answer: The link is created only if your neighbor accepts it.
8. Suppose that your *real* profile is 3 and you want to have an *apparent* profile of 1. How many points do you need to pay?
   Correct answer: 8 points.
9. Suppose that your *real* profile is 0 and you want to have an *apparent* profile of 3. How many points do you need to pay?
   Correct answer: 12 points.

2 Additional results

The following Fig. S1 shows the average cooperation level for the RR and FR treatments. Moreover, we also show the average cooperative acts of reliable and cheater players, respectively. Figure S2 shows the average cooperation indices (true α, Fig. S2a; observable α, Fig. S2b) for the RR and FR treatments, and for reliable and cheater players.

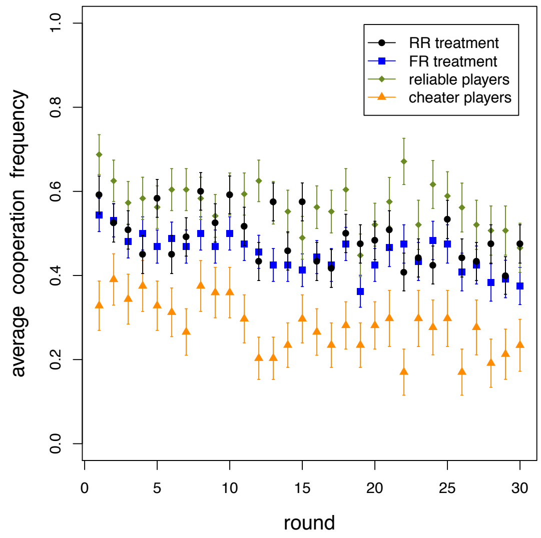

Figure S1: Average cooperation level in the experiment for RR and FR treatments and for reliable and cheater players in the latter case. Error bars represent standard errors of the mean. The difference between final mean values of cooperation level for reliable and cheater players is statistically significant [first repetition, $P^* = 0.046$; both repetitions, $P^{**} = 0.002$]. Other differences are not statistically significant.

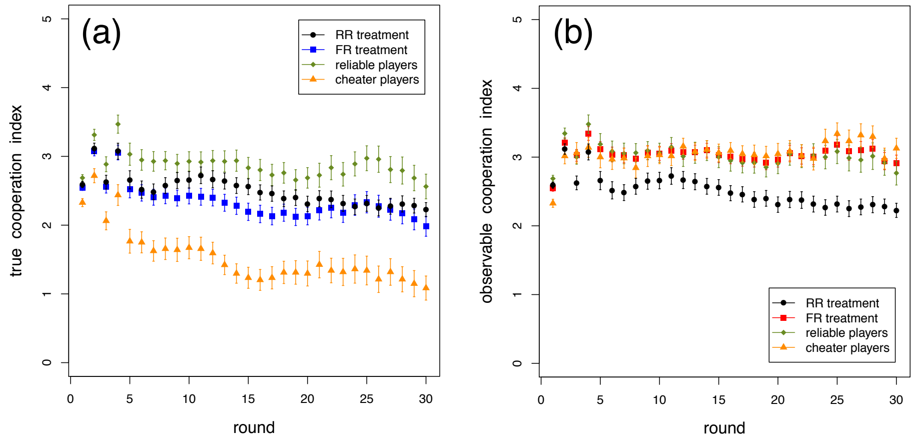

Figure S2: (a): time evolution of the true cooperation index in the whole population after aggregating all the treatments in the baseline case (RR, black dots), the fake reputation treatment (FR, blue squares), and for reliable and cheater players (green squares and orange triangles). (b): time evolution for the observable cooperation index for the same cases. Error bars represent standard errors of the mean. The difference between final mean values of true cooperation index for reliable and cheater players is statistically significant [first repetition, $P^* = 0.036$; both repetitions, $P^{***} < 0.001$]. The difference between final mean values of observable cooperation index for RR and FR treatment is statistically significant considering both repetitions [first repetition, $P = 0.138$; both repetitions, $P^* = 0.019$]. Other differences are not statistically significant.

In Fig. S3 we show the average participants' frequency of cooperation in deciles for the RR treatment (black bars) and for the FR treatment (blue bars). Interestingly, it can be seen that in the RR treatment about one third of the participants cooperate between 50% and 60% of the times. Such a peak of cooperation is not observed in the FR treatment where the frequencies tend to be more uniform. In fact, in the FR treatment some participants decided to maintain a lower cooperation frequency and to increase their observable cooperation index paying the cost.

The following Fig. S4 depicts the evolution of the average degree in the dynamic networks in RR and FR treatments and includes reliable and cheater players. The network mean degree tends to increase but it stabilizes and the graph never becomes a complete one. An interesting effect is that cheaters tend to attract more neighbors in the network since their observable cooperation index is usually higher than the one of reliable players. We note that, for technical reasons, two groups of the FR treatment only performed 20 additional rounds in the second session of the same treatment. All averages have been computed taking this into account.

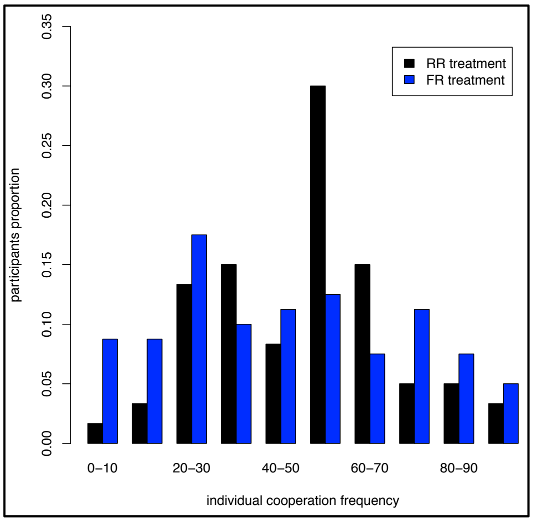

Figure S3: Proportion of participants (y-axis) who have had a given frequency of cooperative acts during the whole experiment (x-axis), cumulated over all sessions and grouped in deciles, for the RR and FR treatment.

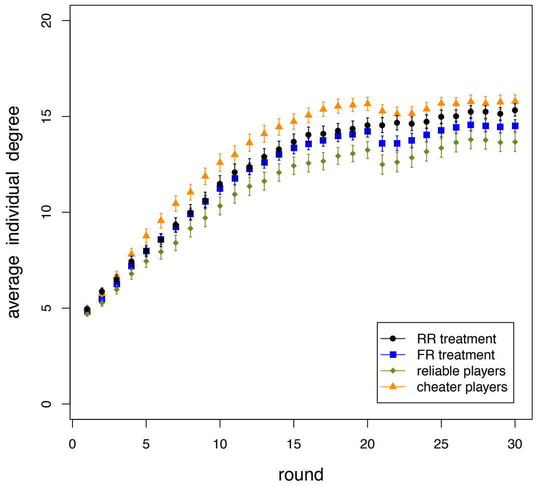

Figure S4: Evolution of the average degree for the RR and FR treatments (all sessions cumulated) and for reliable and cheater players for all sessions in the FR treatment. Error bars represent the standard error of the mean. The difference between final mean values of individual degree for reliable and cheater players is statistically significant [first repetition, $P^* = 0.046$; both repetitions, $P^{**} = 0.010$]. Other differences are not statistically significant.

Figure S5 reports the time evolution of the average payoff and it shows that participants gain more in the RR treatment than in the FR treatment at all times. Conversely, we see that it pays to be a cheater with respect to a reliable player in the FR treatment.

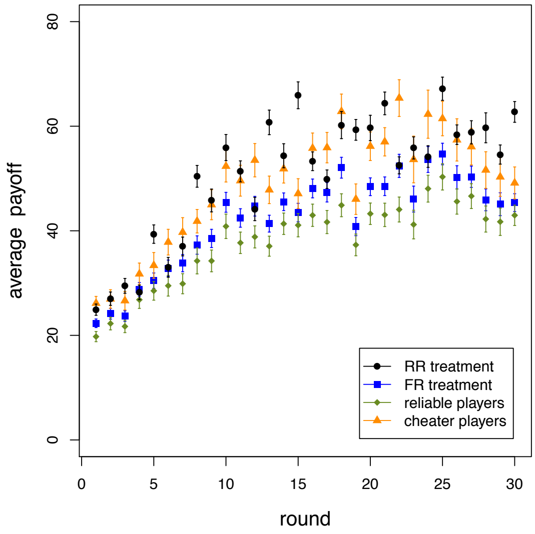

Figure S5: Average payoff per round in the baseline (RR) and in the fake reputation (FR) treatment respectively. We also plot average payoff per round in the FR treatment for reliable and cheater players respectively. Cost is taken into account in these figures. Error bars represent the standard error of the mean. The difference between final mean values of individual payoff for RR and FR treatments is statistically significant considering both approaches [first repetition, $P^* = 0.072$; both repetitions, $P^* = 0.013$]. Other differences are not statistically significant.

Subsequently, Fig. S6 summarizes the main features of individual players by plotting them in a scatterplot given by their link cutting frequency and their cooperativeness in the RR and FR treatment, indicating whether they are reliables or cheaters in the FR treatment. Finally, Fig. S7 depicts the number of accepted proposals in the first stage of the experiment, namely when our experimental setup offers subjects a new link with a randomly chosen player, for the RR and FR treatments.

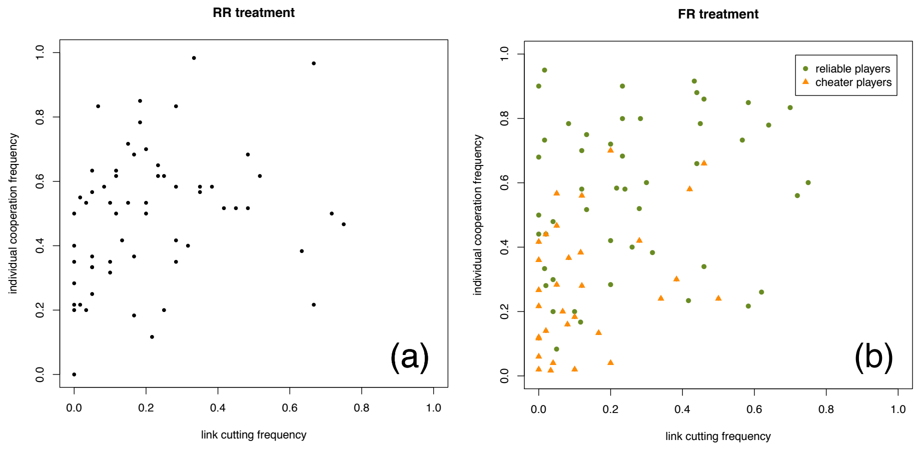

Figure S6: Scatterplots of the players as described by their link cutting frequency (x-axis) and their cooperation frequency (y-axis). Each point corresponds to an individual player. (a) RR treatment. (b) FR treatment. Colors in the FR treatment indicate whether a player is a reliable (green dots) or a cheater (orange triangles).

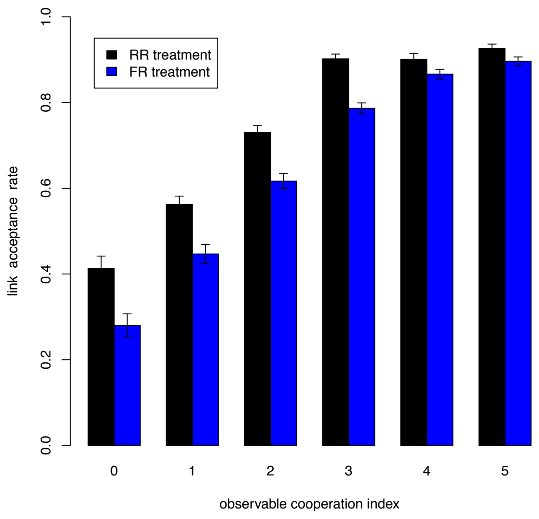

Figure S7: Link acceptance rate at the first stage of the modifications to the network in the experimental setup, i.e., fraction of randomly proposed new neighbors that are accepted as a function of their observable cooperation index. Black bars correspond to the RR treatment, blue bars to the FR treatment. Error bars represent standard error of the mean.

In Fig. S8 we show the normalized number of participants who purchase points (at least one) per round and per participant type in the first repetition of FR treatments. We can see that the vast majority of cheater players fake their cooperation index almost at every interaction, with a particular increase during last rounds. On the other hand, reliable players, by definition, purchased less reputational points and they consequently have a smaller rate.

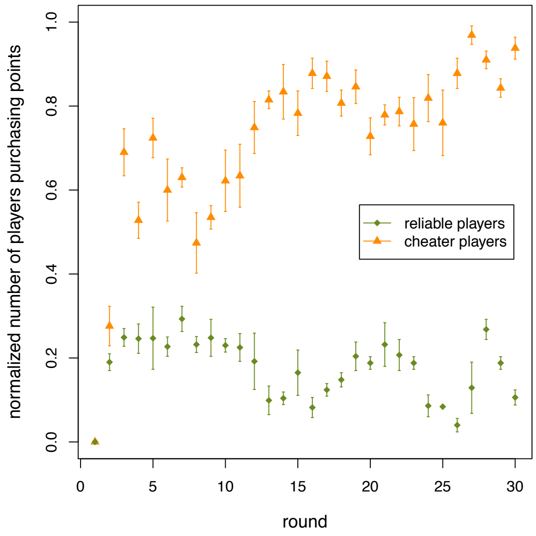

Figure S8: Average normalized number of players who purchase points (at least one) per round and per participant type considering only the first repetition of FR treatments. Error bars represent standard error of the mean.
